# Hematoma expansion prediction based on SMOTE and XGBoost algorithm

**DOI:** 10.1186/s12911-024-02561-9

**Published:** 2024-06-19

**Authors:** Yan Li, Chaonan Du, Sikai Ge, Ruonan Zhang, Yiming Shao, Keyu Chen, Zhepeng Li, Fei Ma

**Affiliations:** 1 Department of Mathematics and Physics, Xi’an Jiaotong-Liverpool University, Suzhou, China; 2 Department of Neurosurgery, Affiliated Jinling Hospital, Medical School of Nanjing University, Nanjing, China

**Keywords:** Hematoma expansion, XGBoost, SMOTE, Machine learning prediction, Unbalanced dataset

## Abstract

Hematoma expansion (HE) is a high risky symptom with high rate of occurrence for patients who have undergone spontaneous intracerebral hemorrhage (ICH) after a major accident or illness. Correct prediction of the occurrence of HE in advance is critical to help the doctors to determine the next step medical treatment. Most existing studies focus only on the occurrence of HE within 6 h after the occurrence of ICH, while in reality a considerable number of patients have HE after the first 6 h but within 24 h. In this study, based on the medical doctors recommendation, we focus on prediction of the occurrence of HE within 24 h, as well as the occurrence of HE every 6 h within 24 h. Based on the demographics and computer tomography (CT) image extraction information, we used the XGBoost method to predict the occurrence of HE within 24 h. In this study, to solve the issue of highly imbalanced data set, which is a frequent case in medical data analysis, we used the SMOTE algorithm for data augmentation. To evaluate our method, we used a data set consisting of 582 patients records, and compared the results of proposed method as well as few machine learning methods. Our experiments show that XGBoost achieved the best prediction performance on the balanced dataset processed by the SMOTE algorithm with an accuracy of 0.82 and F1-score of 0.82. Moreover, our proposed method predicts the occurrence of HE within 6, 12, 18 and 24 h at the accuracy of 0.89, 0.82, 0.87 and 0.94, indicating that the HE occurrence within 24 h can be predicted accurately by the proposed method.

## Introduction

Spontaneous intracerebral hemorrhage (ICH) is defined as sudden bleeding from the brain parenchyma that may extend to the ventricles or subarachnoid space [1]. It has been recognized as an important health issue, contributing to 7.1 million cases and 3.1 million deaths in 2021 [2]. Besides the high mortality rate, ICH may lead to various disabilities, such as epilepsy, psychosis, mood disorders, hemiplegia, and more than 60% of patients can’t regain functional independence [3]. In China, there are 0.6–0.8% of people suffering from ICH every year, and the mortality in the acute stage is between 30 and 40% [3]. Moreover, it is believed that the gravity of the situation will worsen in the future [4] due to the growth of aging population in China. Although medical technology has achieved significant advancements, an effective and safe treatment for ICH has not been performed. Hematoma expansion (HE) is one of the common and significant phenomena of ICH, which is closely associated with the deterioration of early neurological function, and is also an independent predictive factor for poor prognosis and increased mortality.

Sato et al. [5] believe that HE has important prognostic value for adverse outcomes and mortality in patients. In clinical practice, it is crucial to predict whether there is expansion of a cerebral hematoma, and then select the appropriate clinical treatment plan. The hematoma volume is recognized as a focus for the 30-day mortality, and preventing the hematoma from expanding is essential for ICH treatments, such as INTERACT, ATACHII and STOP-AUST [1]. In addition, it is known that early treatments on preventing HE can decrease the death rate of ICH. For example, in 2009, Anderson et al. found that early intensive blood pressure-lowing treatment can limit hematoma growth over 72 h in ICH [6]. However, the optimal time for treatment may slip away because the diagnosis of coagulopathy, such as Prothrombin Time (PT), International Normalized Ratio (INR), Activated Partial Thromboplastin Time (APTT), requires 1 to 2 h to be confirmed after blood sample collection and therefore, it is necessary to develop efficient methods to predict HE.

It was considered that HE tends to occur within 6 h after ICH [7], but in clinical, a considerable number of patients experience HE within the first 24 h after onset. Non-contrast computer tomography (CT) is the preferred initial examination in emergency after onset due to its speed and convenience. Several studies have found that some CT image markers, such as shape and heterogeneity of hematoma, island sign, satellite sign, blend sign, black hole sign and swirl sign, have a significant impact on the prediction of HE within 6 h after onset of ICH [8–10]. These CT image markers were generally considered to be associated with HE within 6 h. The value of these markers in the first 24 h after onset is still uncertain. Therefore, it is necessary to explore whether these markers remain predictive within 24 h of ICH onset.

In recent years, the volume of medical data has exploded, and the development of artificial intelligence has made it possible to analyze and interpret different types of medical information [11]. The use of large models to analyze medical images and electronic medical records to assist doctors in diagnosis and decision-making is becoming increasingly popular. In the healthcare domain, machine learning (ML) algorithms play a major role in accurately classifying and predicting various diseases. With ML, experts can analyze and evaluate datasets containing diagnostic information, electronic medical records, and image information to help them de- velop effective treatment strategies [12, 13]. In this study, we aim to predict HE occurrence within 24 h, establish an effective ML-based prediction method, and verify if the factors used for predicting HE occurrence within 6 h are still applicable for predicting HE occurrence within 24 h. In the following, we will list the main contributions of our work:

- We use the Extreme Gradient Boosting (XGBoost) algorithm to predict the HE occurrence within 24 h, as well within 6, 12, 18 and 24 h.
- We use the Synthetic Minority Oversampling Technique (SMOTE) to process and to cope with the imbalanced dataset.
- We identify the key indicators that contribute to the occurrence of HE based on SHapley Additive exPlanations (SHAP) values.
- Through comparison of results with few state of the art methods, including Support Vector Machine (SVM) [1], Random Forest (RF) [2], Logistic Regression (LR) [14] and k-nearest neighbors (KNN) [15], XGBoost showed better predictive performance, verified that XGBoost can be used for HE occurrence prediction.
- We prove that HE can be accurately predicted within 24 h based on indica-tors.

The rest of the paper is designed as follows: In Sect. 2, we present a literature review of the different methods and results of HE prediction. Section 3 includes dataset description and principle of the methodology. Section 4 and Sect. 5 provide results and discussions respectively, while the last section gives the conclusion.

## Related work

Extensive prognostic scoring systems have been proposed in multiple literature for prediction of HE in ICH, such as A 9-point prediction score which selects warfarin medication history, point signs, time from symptom onset to first head CT, and baseline hematoma volume as evaluation criteria [16], 24-point BRAIN score [17], Hematoma Expansion Prediction (HEP) score [18], 7-point prediction score choosing baseline hematoma volume, mixed sign, island sign, whirlpool sign, anticoagulant therapy, ICH, time from symptom onset to first head CT, and baseline hematoma volume as evaluation indicators [19], HEAVN score [20], NAG scale [21]. In terms of indicator selection, Nawabi [22] employed Cohen’s kappa coefficient for confirming the reliability of CT features on CTA in patients with ICH.

The logistic regression model, as a basic model for predicting dichotomies in statistics, has been widely used in the medical field especially for predicting HE in ICH. Chan et al. [14] used univariate feature selection methods for feature selection, Fisher’s exact test and the Kruskal-Wallis test for each feature to determine the optimal subset of features and multivariate logistic regression to establish an automatic prediction model for HE. In studying 118 patients with ICH, Sakuta et al. [21] utilized univariate feature selection methods such as cardinality test, Fisher exact test, T-test and Mann-Whitney U-test for feature selection. After determining the optimal subset of features, a prediction model was developed using multivariate logistic regression and a scale was created. Besides, Yang et al. [23] performed univariate and binary logistic regression analysis, screened out independent pre- dictors significantly related to HE, and established a new SICH-HE model. This model offers a theoretical foundation for clinicians to promptly identify high-risk HE patients and validate the early surgical decision-making process.

ML has been widely applied in medicine [24] and especially, SVM, RF, DT, KNN and Adaboost all present good performance in predicting HE using routinely available variables [25]. Liu et al. [1] established a SVM model to predict HE, but in comparison to SVM, the model based on the RF algorithm demonstrated higher accuracy [2]. Furthermore, A multi-task deep learning approach that allows simultaneous tumor segmentation and response prediction has two Siamese sub-networks joined at multiple layers, which enables integration of multi-scale feature representations and in-depth comparison of pre-treatment and post-treatment images [26]. Ma et al. [27] compared the prediction effects of ResNet-18, ResNet-34 and VGG- 16 neural networks. ResNet-34 achieves the most robust generalization capability in HE prediction and is superior to other mainstream models, which will facilitate accurate, efficient, and automated HE prediction. It addresses the limitations of neural networks in predicting HE through quantitative volume and texture analysis (CTTA) of CT images [28]. A fuzzy C-means (FCM) intelligent segmentation algorithm was established by Xu et al. [29] for intelligent segmentation of patients’ brain CT images, which holds high clinical value for the early prediction of HE in patients with ICH.

XGBoost, a gradient boosting learning model, has been widely used for analyzing medical data for classification and prediction in healthcare. It has achieved accurate prediction in hypertension outcomes [30], diabetes [31], cardiovascular [32] and coronary heart diseases [33]. Compared with deep learning models, the biggest advantage is that it has faster speed and stronger robustness when processing large-scale datasets [34]. However, the literature on the prediction of HE by XGBoost is scarce. One possible explanation is that the prediction of HE is usually typically reliant CT images. Unless the corresponding indicators are extracted from the images, deep learning models are often more applicable for image data based prediction. Once the image information is extracted and converted into tabular data, XGBoost could also achieve excellent prediction results in HE prediction [35].

SMOTE, a Synthetic Minority Oversampling Technique, is an enhanced method based on the random oversampling, which simply duplicates samples to increase minority samples. SMOTE addresses the issue of model overfitting, this occurs when the model learns overly specific information that lacks generalization [36]. In clinical data analysis, there is often a bias in the data obtained, which means that the ratio of positive data to negative data is not balanced [37]. Therefore, SMOTE has a wide range of applications in the medical field. For instance, Alghamdi et al. [38] used SMOTE to address the negative impact of imbalanced categories in the constructed model when they carried out the project of predicting diabetes. Pandey and Janghel [39] used SMOTE technique to address the issue of class imbalance in the MIT-BIH database for their study on arrhythmia detection. Besides, Wang et al. [40], Francis, Prasad and Zahoor-Ul-Huq [41] and Xu et al. [42] all used SMOTE to solve the problem of uneven data distribution and compared it with other methods, further showcasing the viability and effectiveness of this approach in medical applications.

## Materials and methods

### Population

In this study, we investigated a database of brain hemorrhage cases collected by the emergency department and neurosurgery department of a local hospital in Xuzhou, China. A total of 892 patients diagnosed with ICH from 2014 to 2019 were extracted from the database. All personal information about the patients was erased.

The studied population should met revised diagnostic criteria raised in the 4th National Conference on Cerebrovascular Disease: (1) ICH diagnosed by CT images; (2) Previous history of hypertension; (3) Age no less than 18 years old; (4) First cranial CT within 6 h of onset, and follow-up cranial CT within 24 h of first cranial CT. After applying these criteria, a total of 582 records were retained and used in the study.

#### Data extraction

The patient’s cranial imaging findings were interpreted by the imaging physician to determine the site of the cerebral hemorrhage, the volume of the hematoma, whether the hematoma was regular, and whether the hematoma had broken into the ventricles. If the absolute value of the hematoma volume increased by 6 ml or the percentage of increase in the hematoma volume was above 33% between the first and second CT examinations, the hematoma was considered enlarged. The hematoma was divided into two groups: the HE group (case group) and the non-HE group (control group).

The demographics and CT image extraction information we used as indicators in this study included age, gender, diabetes mellitus, alcohol use, ICH position,  adimission GCS score, baseline hematoma volume and hematoma expansion, as well as admission SBP, and admission DBP, left or right, hematoma shape score, hematoma heterogeneity, island sign, satellite sign, black hole sign, blend sign, swirl sign, IVH, SAH and MLS. Among those indicators, hematoma expansion is our target predictive variable. Table 1 lists and describes these indicators.

**Table 1:** List of abbreviations

Abbreviations	Full Name
SBP	Systolic blood pressure
DBP	Diastolic blood pressure
IVH	Intraventricular hemorrhage
SAH	Subarachnoid haemorrhage
MLS	Medical laboratory science
GCS	Glasgow coma scale
Left/Right	Site of ICH

#### Statistics

Of these 582 patients studied, 114 (19.6%) of them were diagnosed with an HE. The composition for the different variables are shown in Table 2. Univariate analysis shows the differences between the two groups are statistically significant in terms of admission SBP, admission DBP admission GCS score and baseline hematoma volume (p *<* 0.05). While the other indicators are not statistically significant (p *>* 0.05).

#### Data pre-processing

Classification variables, such as gender and various signs, were transformed into binary variables by label encoding. In terms of gender, male was encoded as 1 and female was encoded as 0. In terms of various signs, those that appear were encoded as 1, while those that did not appear are coded as 0. A special variable is the ICH position. Given that almost 50% of ICH occurs in the basal ganglia, we group the basal ganglia into one category, and uniformly assign the remaining positions to another category for coding. After the feature standardization is complete, we fill in the missing values with the average value of the column. This step is specific to machine learning models except XGBoost, which has its own processing for missing values. For all continuous variables, we kept the values and applied Z-score normalization to the data.

**Table 2:** Variable description

Characteristics	Case group	Control group	*P* value
Age	59.28±13.51	60.12±12.41	0.03
Gender (Male)	53(46.5%)	221(49.5%)	0.58
Diabetes mellitus	9(7.9%)	64(15.1%)	0.19
Alcohol use	12(10.5%)	43(9.4%)	0.19
Admission SBP	173.63±20.57	168.63±19.24	0.02
Admission DBP	101.25±14.39	98.56±12.31	0.04
ICH position	79(46.8%)	196(41.8%)	0.56
Left/Right	62(47.7%)	182(38.9%)	0.45
Shape score	2.81±1.63	2.80±1.61	0.34
Heterogeneity	3.72±1.68	3.64±1.59	0.43
Island sign	45(39.5%)	112(23.9%)	0.28
Satellite sign	10(8.8%)	45(9.6%)	0.24
Black hole sign	15(13.2%)	47(15.2%)	0.17
Blend sign	60(52.6%)	192(46.8%)	0.12
Swirl sign	13(11.4%)	52(12.8%)	0.61
IVH	39(34.2%)	107(39.7%)	0.54
SAH	7(6.1%)	22(7.2%)	0.77
MLS	34(29.8%)	102(28.8%)	0.89
Admission GCS score	10.49±2.99	10.27±2.21	0.19
Hematoma volume	15.73±15.26	14.78±12.37	0.01

The first column corresponds to the name of the indicators used, and the second and third columns correspond to the statistics of each indicator in the case group and the control group, respectively. The statistic of numerical indicators such as age is expressed by means variance, and the statistic of binary indicators such as alcohol use is expressed by number and ratio. P values in the last column are used to determine whether various indicators are significant

### Implementation process

After obtaining the preprocessed data, we first randomly split the dataset into a training set and a test set in the ratio of 8:2. We then applied a variety of machine learning algorithms to train on the training set and validate on the test set, and compared the accuracy, precision, recall and F1-score obtained by different algorithms. Meanwhile, the parameters of the algorithms were determined by the Grid Search in Sklearn. Afterward, we used SMOTE to augment the data for the case group, ensuring that its sample size was the same as the control group. The same process was used to train the augmented data, and the predicted performance was combined and discussed with the results previously obtained. After training the models, we analyzed the indicator importance using SHAP value.

**Fig. 1:**
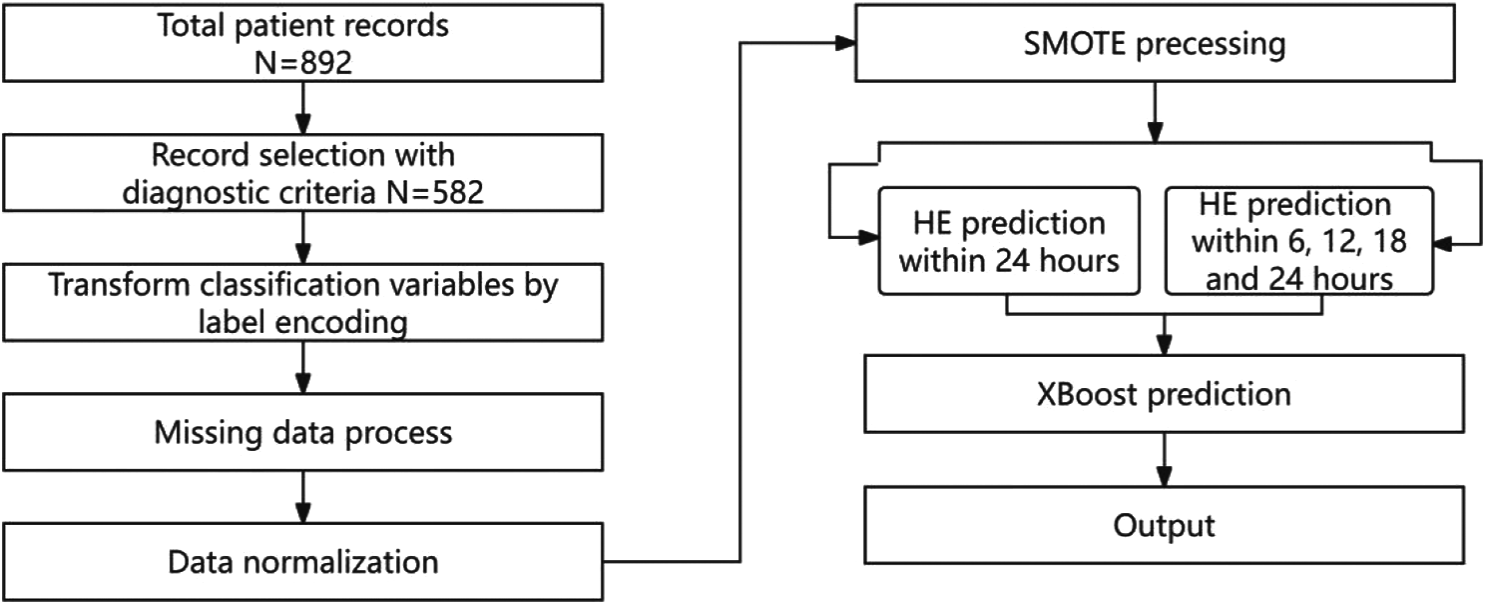
Steps of the proposed model

In addition, we divided the augmented data into four groups according to the time interval between the first and subsequent CT examinations. We demonstrated that HE can be accurately predicted within 24 h based on indicators by com- paring the predictive performance among the four groups. The complete steps of our proposed model are presented in Fig. 1.

### XGBoost prediction

Extreme Gradient Boosting (XGBoost) is a reliable and open-source gradient tree boosting model. It started as a research project by Tianqi Chen in 2014 [43]. As a supervised learning algorithm, it combines an ensemble of estimates from a set of trees. Compared to traditional gradient boosting decision trees, XGBoost has the advantage of column sampling and can also continue tree construction with missing values by transforming the missing values into a sparse matrix, which can effectively helps avoid some overfitting problems.

Given a dataset of form:

$${\cal D} = \{ ({x_i},{y_i}):i = 1...n,{x_i} \in {{\bf{R}}^m},{y_i} \in {\bf{R}}\}$$,

we get *n* observations with *m* features each and with a corresponding variable *y*. Let ${\hat y_i}$ be defined as a result given by an ensemble represented by the generalised model as follows:

1 $${\hat Y_i}\; = \;\sum\limits_{k = 1}^K {{f_k}} \left( {{x_i}} \right)$$

In the above formula, ${f}_{k}$ is a regression tree, and ${f}_{k}\left({x}_{i}\right)$ represents the score given by the k-th to the i-th observation in data.

Then the objective function to be minimized in step t is expressed as:

2 $${L^{\left( t \right)}} = \sum\limits_{i = 1}^n l ({y_i},\;\hat y_i^{(t - 1)} + {f_t}({x_i})) + \omega \left( {{f_t}} \right)$$

where $\hat y_i^{(t - 1)}$ denotes the prediction result of the previous $t-1$ trees for sample ${x}_{i}$, ${f}_{t}$ stands for the t tree, $l$ is loss function and $\omega$ is the canonical term used for the t-th tree [44].

### Smote

An imbalanced dataset is one in which the number of examples in one class is significantly different from the number of examples in other classes. To deal with the over-fitting problems that often occur when facing an imbalanced datasets, a Synthetic Minority Oversampling Technique called “SMOTE” was proposed by Chawla et al. [36]. The fundamental concept of this method is to generate new samples for the minority class in the data set by means of a linear interpolation algorithm. Compared with random over-sampling techniques, this algorithm can increase the variety of training samples instead of repeating the original training samples, thus effectively solving the over-fitting problem. The steps for this technique are described as follows [45]:

a) For each sample point ${x}_{i}$ in the minority class set A, calculate its Euclidean distance with every other points in set A, and obtain the k-nearest neighbours of ${ x}_{i}$.

b) For k-nearest neighbours of *x*_*i*_, arbitrarily choose the appropriate number of samples *N* (i.e. ${\text{x}}_{1}$,…,${\text{x}}_{\text{N}}$) to form a new sample set *A*_1_. Here the sampling multiplier N is based on the proportion of sample imbalance.

c) For every ${x}_{j}\in {A}_{1}$ (${j}=1,2,...,N$), A new sample point ${x}_{i}$ is synthesized by the following linear interpolation formula:

3 $${x}_{i}^{{\prime }}={x}_{i}+rand\left(\text{0,1}\right)\cdot |{x}_{i}-{x}_{j}|$$

d) The newly generated minority samples are combined with the original sample A to form a new sample A′ for training.

In this study, the number of patients with HE was much lower than the number of patients without HE. The imbalance in the data samples significantly affects the performance of the prediction model, therefore, before constructing the prediction model [46], we propose balancing the dataset using the SMOTE algorithm, which can generate new minority class samples to achieve a balanced data sample between classes. The distribution of the data before and after SMOTE can be seen in Table 3.

**Table 3:** Samples before and after using SMOTE

Class	1–6 h		7–12 h		13–18 h		19–24 h	
	Before	After	Before	After	Before	After	Before	After
Class 0 Non Hematoma Expansion	117	117	103	103	117	117	155	155
Class 1 Hematoma Expansion	29	117	27	103	28	117	28	155

### SHAP values

One of the major problems with machine learning models is that the models themselves are not interpretable, and SHAP (SHapley Additive exPlanations) is one approach to tackle this problem. SHAP is based on the Shapley value, a game-theoretic concept introduced by economist Lloyd Shapley, which is interpreted by SHAP as an additive feature attribution method that determines the importance of an individual by calculating the contribution of that individual in cooperation. The model explains the predicted values through a linear function of binary variables [47]:

4 $$g\left(z\right)={\varphi }_{0}+\sum _{i=1}^{M}{\varphi }_{{z}_{i}}$$

Here ${\varphi }_{0}$ stands for the the typical prediction, $M$ is the number of features of the simplified input and the SHAP value ${\varphi }_{{z}_{i}}$ represents its direct effect on the model prediction.

We calculate the SHAP value for each of the covariates on the test set. After that, a summary plot was drawn to present the SHAP values of each feature, and by colour we can see the relationship between the size of the feature and the predicted impact, as well as showing its eigenvalue distribution. Meanwhile, the dependence plot clearly shows how individual features affect the prediction results of the model.

## Results

### Predicted performance

Overall, the five models produced better results in terms of precision, recall and F1- score with the balanced datasets (see Table 4). However, the accuracy of the models with the balanced datasets was lower than with the imbalanced dataset, which indicates that the classification of HE was towards the majority samples of non-HE. These results clearly show that designing models using imbalanced datasets will lead to significant inaccuracies, which cannot identify HE and non-HE precisely and this verifies the necessity of using a balancing algorithm to balance datasets in the first step of the classification process. In contrast, the F1-score is more convincing when evaluating a model’s predictive performance on unbalanced data.

In addition, the ensemble models outperformed the single classifiers, as determined by the performance indicators, among which, the Area Under Curve and precision values of XGBoost with SMOTE exceeded those of SVM, RF and LR with SMOTE algorithm. Particularly, XGBoost with SMOTE produced the highest results among all classifier models with an accuracy of 0.82 and a F1-score of 0.82 on a balanced dataset. Especially, the F1-score value indicates that the XGBoost model can distinguish between HE and non-HE precisely.

**Table 4:** Predictive performance of different models on balanced and imbalanced datasets

Dataset	Methods	Accuracy	Precision	Recall	F1-score
IBT	XGBoost	0.90	0.51	0.62	0.60
IBT	SVM [1]	0.87	0.44	0.50	0.47
IBT	RF [2]	0.85	0.43	0.46	0.49
IBT	LR [14]	0.86	0.44	0.50	0.46
IBT	KNN [15]	0.87	0.44	0.51	0.48
BT	XGBoost	0.82	0.82	0.82	0.82
BT	SVM [1]	0.78	0.81	0.77	0.77
BT	RF [2]	0.80	0.79	0.77	0.80
BT	LR [14]	0.69	0.69	0.69	0.69
BT	KNN [15]	0.80	0.78	0.77	0.78

The first five rows correspond to the predictions of the five machine learning algorithms on the imbalanced dataset, and the last five rows correspond to their respective predictions on the balanced dataset.

### Feature importance

**Fig. 2:**
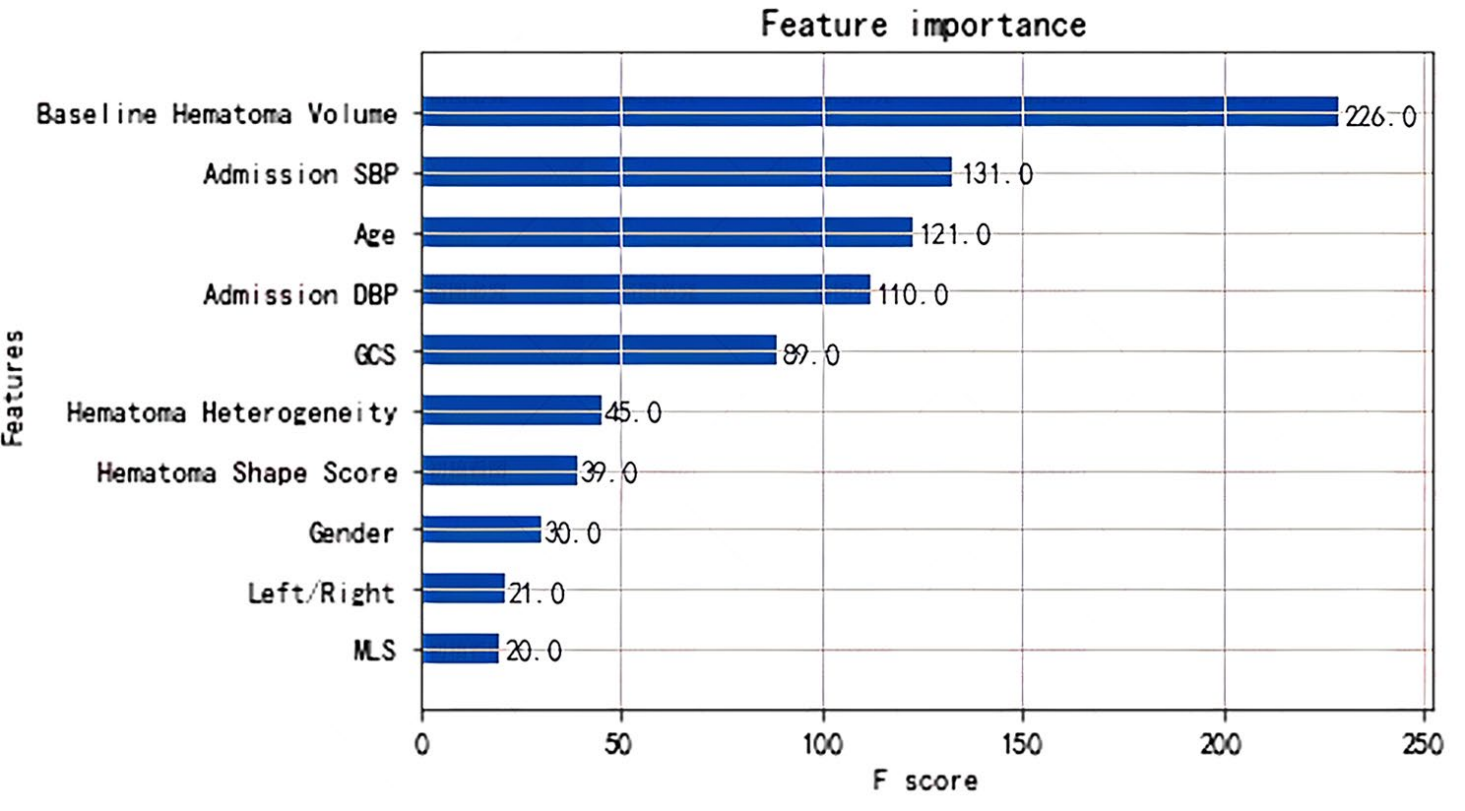
Contributions of features from XGBoost for the whole dataset

Figure 2 shows the list of the top 10 features among all that are used in the XGBoost model, following the order of contribution for each evaluation metric. Of all, the most influential covariate for predicting whether a hematoma will enlarge is the initial hematoma volume. Admission SBP, age and admission DBP also play an important role in forecasting.

### SHAP value analysis

**Fig. 3:**
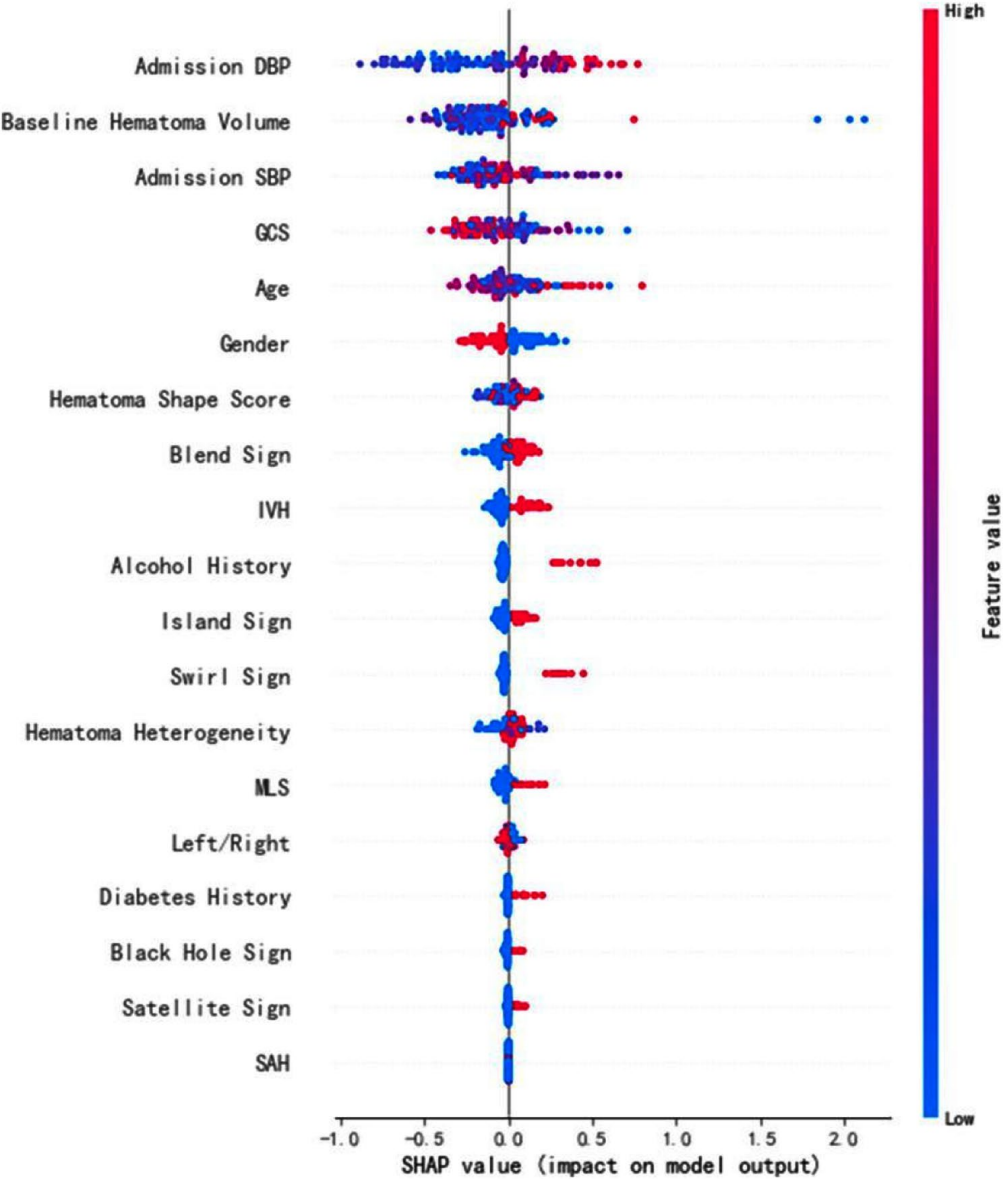
SHAP value (effect on model output)

We analyzed the relative effect of the top 10 features on the model at each data point in the test set according to the mean absolute SHAP value (Fig. 3). The summary plot was applied to identify influential covariates. Each point in the summary plot indicated the Shapley value and observation value for the characteristic, with the color indicating the value of the characteristic. According to these results, baseline hematoma volume, admission DBP, age, admission SBP and GCS carried model’s forecasting power.

**Fig. 4:**
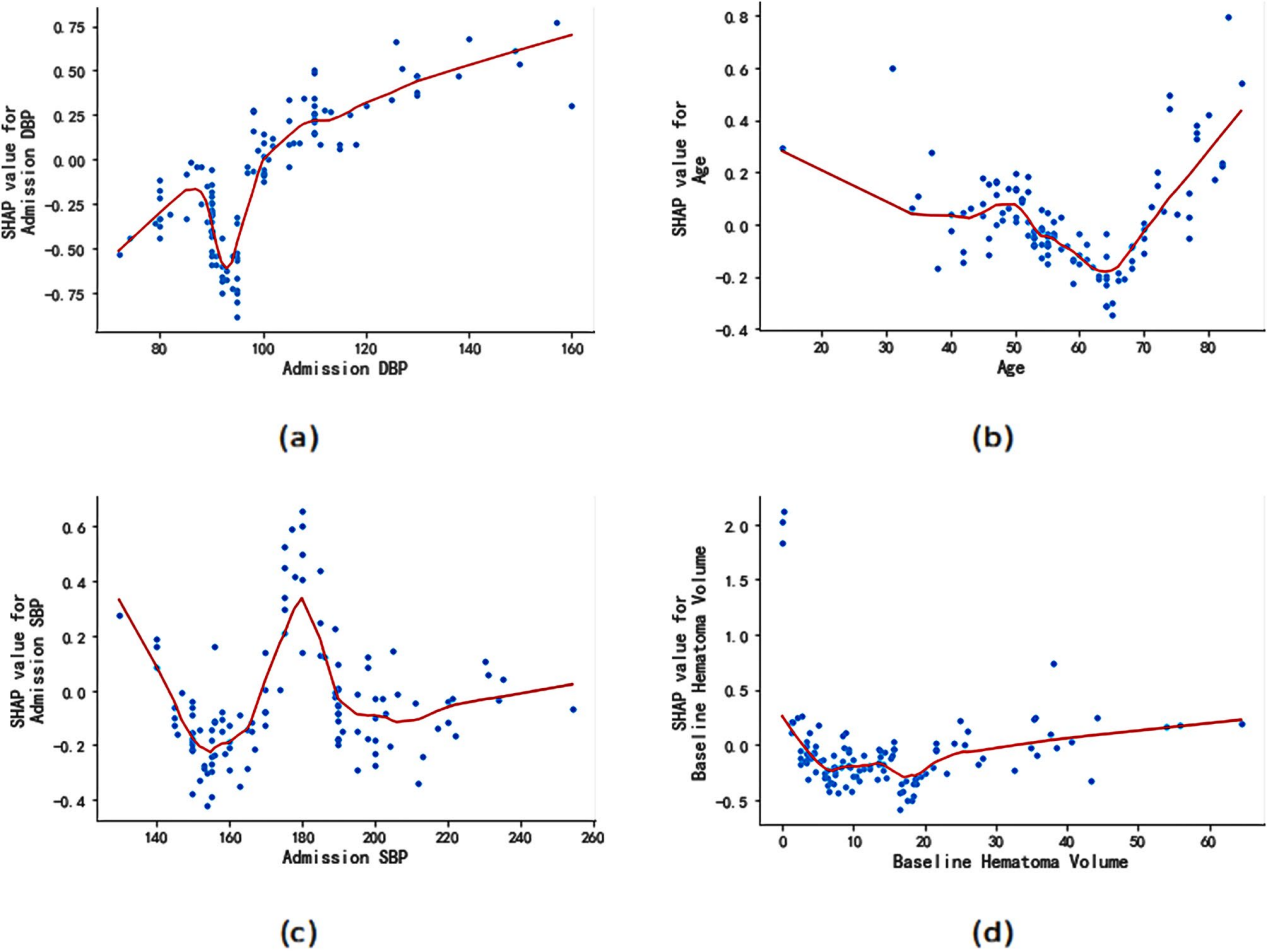
Four examples of dependence plots showing the effect on the HE with respect to the feature value. Points represents the SHAP values, while lines indicate the LOESS fitted smooth representation of the relationship

To further investigate the impact of each variable, we analyzed the SHAP values of the selected 4 important covariates separately in Fig. 4. The admission DBP, except for the range of 85 to 100, positively contributes to the HE (Fig. 4(a)). For age, we saw a relationship with HE before and after 65 (Fig. 4(b)). In terms of admission SBP, the relationship with HE is “W” shaped and the peak of SHAP value occurring when the admission SBP is 180 (Fig. 4(c)). As for the baseline hematoma volume, the overall trend is relatively stable, with the lowest point occurring when the baseline hematoma volume equals 18 (Fig. 4(d)).

### Predicted performance of different time groups

In previous studies [8, 48–50], CT imaging markers such as shape and heterogeneity of hematoma, island sign, satellite sign, blend sign, black hole sign, and swirl sign had good effects on the prediction of HE within 6 h after the onset of ICH. Studies have found that more than one third of ICH patients who underwent CT scanning within a few hours after onset had HE [43]. But there were still a number of patients who could have HE within the first 24 h after 6 h. Thus, we further investigated the predictive ability of these markers to HE within 24 h after ICH. We have evenly divided 24 h into four parts, that is, *T*_1_ = (0, 6], *T*_2_ = (6, 12], *T*_3_ = (12, 18] and *T*_4_ = (18, 24].

Table 5 summarizes the performances of the algorithms in particular time periods *T*_1_, *T*_2_, *T*_3_ and *T*_4_ and it is obvious that the disaggregated results are better than that presented in Table 4. At the same time, XGBoost has also achieved the best prediction performance on different time groups among all machine learning algorithms, whereas LR presented the worst predictive ability in the dataset.

By comparing the predictions between groups, the highest accuracy and F-score was achieved in time group *T*_4_ and the two values were 0.94 and 0.93, respectively. It is worth noting that, except for the first time group, the predictive performances of the models all improve long with time implying that although the area under the curve of ROC curves decreased with time, it still maintained a high accuracy. Therefore, these CT image markers have high predictive power and could be regarded as reliable indicators for predicting HE in the first 24 h after ICH. Besides, our model also validates that HE occurring within 24 h can be predicted with the help of machine learning models.

**Table 5:** The predictive results on different time groups

Methods	Accuracy	Precision	Recall	F1-score
(a) Time group *T*_1_				
XGBoost	0.89	0.91	0.90	0.89
SVM [1]	0.77	0.84	0.76	0.75
RF [2]	0.86	0.89	0.89	0.88
LR [14]	0.74	0.78	0.75	0.74
KNN [15]	0.82	0.88	0.88	0.88
(b) Time group *T*_2_				
Methods	Accuracy	Precision	Recall	F1-score
XGBoost	0.82	0.83	0.83	0.80
SVM [1]	0.70	0.70	0.83	0.69
RF [2]	0.80	0.81	0.81	0.78
LR [14]	0.69	0.69	0.67	0.68
KNN [15]	0.78	0.78	0.77	0.78
(c) Time group *T*_3_				
Methods	Accuracy	Precision	Recall	F1-score
XGBoost	0.87	0.87	0.88	0.87
SVM [1]	0.77	0.82	0.74	0.74
RF [2]	0.85	0.85	0.85	0.85
LR [14]	0.74	0.74	0.75	0.74
KNN [15]	0.86	0.86	0.87	0.87
(d) Time group *T*_4_				
Methods	Accuracy	Precision	Recall	F1-score
XGBoost	0.94	0.94	0.93	0.93
SVM [1]	0.79	0.85	0.80	0.78
RF [2]	0.92	0.92	0.92	0.92
LR [14]	0.84	0.84	0.84	0.84
KNN [15]	0.92	0.92	0.92	0.92

## Discussion

This study has developed classification models to forecast HE based on different machine learning models combining the SMOTE algorithm. By analysing real cases of cerebral hemorrhage in hypertensive patients over the past six years, we have confirmed the feasibility of such hematoma prediction and summarized the main features for prediction results.

### Risk factor

Many risk factors for HE have been clinically proven. As did previous studies, we found that elevated SBP is a risk factor. Also, age, initial hematoma volume and DBP differed between expanders and non-expanders. In addition, HE can also be induced by an increase in SBP, but in our study, we found that SBP at admission did not show a linear relationship with HE. A higher SBP did not correspond to a higher probability of HE. As this is a retrospective exercise, the data set itself may be subject to selection bias, and therefore, a prospective double-blind study is required. Furthermore, many studies have illustrated that GCS score is the most important risk factor for determining ICH patients. However, in this study, the GCS was not particularly important for the outcome. This argument is also consistent with Rangaraj’s [51] findings.

The current treatment for ICH is mainly in the management approach in reducing SBP value since early antihypertensive treatment can effectively reduce the risk of HE [52, 53]. Studies [54, 55] have shown that intensive blood pressure lowering within 24 h of admission can reduce the risk of HE and thus reduce the risk in patients. The specific criteria for lowering blood pressure are to lower SBP to below 180. As we have discussed regarding Fig. 4(c), the peak of the SHAP value occurs when the admission SBP equals 180. Our findings also validate previous studies.

### Imbalanced data sets

As Dong et al. [56], and Liu [57] pointed out, a common issue in current research in the medical field is how to handle imbalanced datasets. The low prevalence of many diseases results in a small proportion of the data set being labeled with this type of disease. Unfortunately, most machine learning algorithms typically make poor predictions for the minority class. Thus, being able to make accurate predictions for these few occurrences of disease is valuable. When facing such a dataset, resampling the training data is a practical method to address the issue of imbalance. Down-sampling majority classes, over-sampling minority classes, or some combinations are commonly applied [44, 47]. For example, the ratio of the sample size of the experimental and control groups was adjusted from 5:1 to 1:1 by the SMOTE algorithm. The original over-accuracy and under-performance of the F1-score were iterated to obtain a more convincing result and provides a reliable basis for clin- ical clinical applications. Another approach is cost-sensitive learning, which reformulates existing learning algorithms by giving more weight to the minority classes [58, 59].

### Ensemble boosting learning methods

A comparison among different methods in this study clearly demonstrates that machine learning algorithms can achieve more accurate prediction performance than logistic regression algorithms for such multivariate datasets. In particular, ensemble boosting learning methods such as XGBoost, tend to be favored. More studies are experimenting with various ensemble boosting learning methods to predict ICH patients instead of the traditional extensive prognostic scoring system. Ensemble boosting learning algorithms integrate several weak classifiers to reduce the poten- tial bias of individual model, obtaining significantly superior generalization performance compared to a single learner and avoiding the production of biased and unstable results [60, 61].

With the continuous optimization of deep learning, it has proven to be a powerful predictive tool in the field of healthcare prediction. However, in the classification and prediction of tabular data, XGBoost demonstrates a large advantage over deep learning models in terms of both accuracy and time, as evidenced in many literature [34, 62]. In addition, deep learning models are often challenging to interpret, which can lead to doctors distrusting the predictive performance of these models if their predictions contradict their intuitive judgment. In contrast, the classical ML model can depict the effects of different variables on the prediction results through SHAP value, which provides interpretability of the prediction results of the XGBoost model, making it more acceptable to physicians.

### Limitations

First of all, most of our data is derived from CT image findings and lacks clinical testing indicators like routine blood test results. However, these results have also proven to be important in correctly predicting ICH patients [51]. In addition, our training and test sets are unified from a single system and the results are only for this batch of data sets, it would be more ideal to include some external data sets to validate the reliability and robustness of our model.

## Conclusion

HE is a high risky symptom happening frequently on patients who have undergone spontaneous ICH. Correct prediction of the occurrence of HE yields great value towards determination of critical medical treatment. This study developed a prediction model based on XGBoost to forecast the occurrence of HE. In the comparison of the prediction results obtained by our proposed method and few other machine learning methods, our proposed method achieved the best prediction performance with a prediction accuracy of 0.82 on the balanced dataset processed by the SMOTE algorithm. On the predictions of HE occurrence within 6, 12, 18 and 24 h, the accuracy of the predictions with the proposed method all exceeded 0.8. We have confirmed that HE can be accurately predicted within 24 h based on indicators in a retrospective study. Through our study we can conclude that hematoma volume, admission SBP and admission DBP contribute greatly to the occurrence of HE.

It has been presented that machine learning algorithms can effectively integrate diverse medical data to accurately and efficiently predict targets. Future research is directed towards exploring the generalisability of our proposed predictive model and exploring more advanced data generation algorithms. AI techniques, such as generative AI, could be used to create possible training data, without which, some latest AI methods, such as deep learning based methods, cannot be employed due to its need of large training data set. However, accuracy and closeness of the generated data to the real data need to be researched before generative AI generated data can be used for model training.

## Data Availability

The datasets analysed during the current study are not publicly available. Inquiries regarding datasets and codes access can be directed via email to: yan.li14@student.xjtlu.edu.cn.
